# Formation of translational risk score based on correlation coefficients as an alternative to Cox regression models for predicting outcome in patients with NSCLC

**DOI:** 10.1186/1742-4682-8-28

**Published:** 2011-07-27

**Authors:** Wolfgang Kössler, Anette Fiebeler, Arnulf Willms, Tina ElAidi, Bernd Klosterhalfen, Uwe Klinge

**Affiliations:** 1 Institute of Computer Science, Humboldt University, Berlin, Germany; 2 Department of Nephrology and Hypertension, Medical School Hannover, Germany; 3 Surgical Department of the Military Hospital, Koblenz, Germany; 4 Experimental Medicine and Immunotherapy, Institute for Applied Medical Technology, University Hospital RWTH Aachen, Germany; 5 Department of Pathology, Hospital Düren, Germany; 6 Department of Surgery, University Hospital RWTH Aachen, Germany

## Abstract

**Background:**

Personalised cancer therapy, such as that used for bronchial carcinoma (BC), requires treatment to be adjusted to the patient's status. Individual risk for progression is estimated from clinical and molecular-biological data using translational score systems. Additional molecular information can improve outcome prediction depending on the marker used and the applied algorithm. Two models, one based on regressions and the other on correlations, were used to investigate the effect of combining various items of prognostic information to produce a comprehensive score. This was carried out using correlation coefficients, with options concerning a more plausible selection of variables for modelling, and this is considered better than classical regression analysis.

**Methods:**

Clinical data concerning 63 BC patients were used to investigate the expression pattern of five tumour-associated proteins. Significant impact on survival was determined using log-rank tests. Significant variables were integrated into a Cox regression model and a new variable called integrative score of individual risk (ISIR), based on Spearman's correlations, was obtained.

**Results:**

High tumour stage (TNM) was predictive for poor survival, while CD68 and Gas6 protein expression correlated with a favourable outcome. Cox regression model analysis predicted outcome more accurately than using each variable in isolation, and correctly classified 84% of patients as having a clear risk status. Calculation of the integrated score for an individual risk (ISIR), considering tumour size (T), lymph node status (N), metastasis (M), Gas6 and CD68 identified 82% of patients as having a clear risk status.

**Conclusion:**

Combining protein expression analysis of CD68 and GAS6 with T, N and M, using Cox regression or ISIR, improves prediction. Considering the increasing number of molecular markers, subsequent studies will be required to validate translational algorithms for the prognostic potential to select variables with a high prognostic power; the use of correlations offers improved prediction.

## Background

Bronchial cancer, a common malignant tumour in the western world, presents as Non-Small Cell Lung Cancer, NSCLC, in more than 85% of cases [1]. It is the leading cause of mortality in terms of malignant disorders, and its incidence is increasing [2]. The underlying pathology is complex and numerous proteins have been described as prognostic markers, demonstrating altered expression compared with healthy surrounding lung tissue [3]. The expression pattern of epidermal growth factor receptor (EGFR) can determine outcome and is used to influence individual therapy [4,5]. However, only a subset of patients benefit from this specifically targeted therapy because they have a specific mutation. Therefore, marker constellations that predict the risk for recurrence and can aid individual-targeted treatment would be advantageous for the majority of patients. Despite progress in microscopic and molecular analyses, the TNM grading scale, which considers the tumour, nodes and metastases, is still the preferred classification scheme for malignancies [6]. However, growing knowledge concerning several factors that are considered to improve or worsen prognosis has resulted in the medical community facing a major challenge to define the prognostic impact of a patient's individual constellation.

An increasing number of biomarkers that reflect the distinct aggressiveness of tumours have been identified. Therefore, they are assumed to predict a patient's risk of tumour progression. For example, the Carmeliet group recently published results that underline the promoting role of a small protein, growth arrest specific protein (Gas) 6, for tumour metastasis in mice [7]. Previously, McCormack et al. demonstrated that Gas 6 expression was positively correlated with favourable prognostic variables in human breast cancer [8]. An accumulation of tumour associated macrophages (TAM) in the stroma of a tumour may serve as an immunological indicator of the defence capability of a host. However, its consequence for survival may be divergent, promoting a good or bad prognosis [9].

Considering the complex interactions within tumours, it is unlikely that one single marker will be sufficient to predict outcome [10]. Therefore, prediction of prognosis will rely on a combination of numerous clinical data concerning the individual patient, particularly information relating to biomarkers. However, translational integration of this large amount of information into one risk assessment is a major challenge. A multiple regression model derived from available data is the current method used to estimate prognosis for a patient. However, the selection of variables is significantly influenced by the choice of the underlying model [11]. As a possible alternative or supplement, this study employed correlations with survival to select variables, and weighted the individual status of each, resulting in an integrated score for an individual risk (ISIR). The resulting ISIR score should predict the outcome, reflecting the individual balance between significant aggressive and protective factors.

To evaluate ISIR, the course of non-small cell lung cancer (NSCLC) was investigated in 63 consecutive patients. In addition to TNM, the expression of several proteins involved in tumour genesis, particularly Gas6, and the number of infiltrating macrophages (CD68) were analysed. In addition, the proteins Notch 3, MMP2 and COX2, were researched to confirm their roles during chronic inflammation and foreign body responses [12]. Each variable was analyzed individually for its prognostic value and subjected to multiple Cox regression analysis. The potential of the newly developed ISIR to predict outcome was evaluated by calculating receiver operating characteristics (ROC) curves and the area under the curve (AUC). The validity of the model was evaluated using leave one-out cross validation.

## Materials and methods

### Patients

The course of 63 patients with NSCLC who were subjected to an operation between 2000 and 2002 was investigated. The local ethical committee approved the study and written, informed consent was obtained from participants. Clinical data included tumour grading according to TNM, level of resection R, histology, gender and age.

### Immunohistochemistry

Tumour sections were evaluated for histology and protein expression by three independent experts. To characterise the tumour-host interaction, the following antibodies were used: CD68 mouse monoclonal antibody (Dako), Gas6 polyclonal anti-goat antibody (Santa Cruz), Notch3 polyclonal anti-goat antibody (Santa Cruz), Cox2 polyclonal rabbit antibody (DCS Innovative Diagnostic Systems), MMP2 polyclonal rabbit antibody (Biomol). As secondary antibody we used biotinylated goat anti-rabbit for Cox2 and MMP2, goat anti-mouse for CD68, and rabbit anti-goat for Notch3 and GAS 6 (all obtained from Dako).

For semi-quantitative analysis, a grading scale was used: 1 indicated very weak staining (<5% cells), 2 indicated weak (5-30%), 3 specified good (30-80%), and 4 indicated a strong (>80%) staining signal. For each marker, a minimum of five view fields were analyzed.

### Statistics

Simple descriptive statistics were computed for squamous cell carcinoma (SCC) and adenocarcinoma (AC), separately. Tests concerning significant differences between the two groups were carried out using a chi^2 ^test for homogeneity and Fisher's exact test. For age and survival, nonparametric confidence intervals were calculated.

Each marker was considered in isolation and Kaplan-Meier curves for the various realizations were generated. Furthermore, log-rank tests were performed to compare survival times. Spearman correlation coefficients between survival and the various variables were computed; a p-value < 0.05 was considered significant. All variables with significant negative or positive correlations to survival time were selected for calculation of the ISIR.

Denoting the significant aggressive variables by *x_i_, i *= 1, ..., k_1 _, the protective variables by y_j_, j = 1,..., k_2 _, and the survival time by t, the numerator of ISIR was defined as the negative of the weighted average  of the aggressive variables, where the weights r_S_(x_i_, t) were given by the Spearman correlation coefficients with the survival time. Similarly, the denominator was defined as the weighted average  of the protective variables,

Inserting the realizations of the variables for any patient resulted in an individual ISIR score, with large values for ISIR indicating high risk.

For the evaluation of ISIR a classification table of prognosis was computed and, as reported by Chen et al., three survival groups were defined: ≤ 12, between 12 and 60, and ≥ 60 months [13]. Furthermore, three ISIR classes were defined, where ISIR ≤ 0.25 denotes low risk, ≥ 0.5 high risk, and ISIR between 0.25 and 0.5 intermediate risk. The Spearman correlation of ISIR to survival was calculated, and scatter plots of the two variables were retrieved. Classification tables were computed with estimates of the sensitivities and specificities. Integrating all features of interest into ISIR, the fact that the different variables have different scale measures (0 to 3 for N, 1 and 2 for M and H, 1-4 for the other) had to taken into consideration. Therefore, each variable was divided by the number of their possible realizations (i.e. by two for M and H, by four for the others).

To emphasize the power of ISIR, it was compared with the well-established Cox method. In Cox regression, we have the so-called proportional hazards model (the Cox model) *λ*(*t*,**X**) = *λ*_0_(*t*)exp(**X*β***), where *λ*(*t*,**X**) is the hazard rate at time point *t *and with given vector **X **of covariates. The baseline hazard and *λ*_0_(*t*) the vector ***β ***of regression coefficients are estimated. It is very common to use automatic backward variable selection, and variables are removed from the model when p > 0.05.

The statistical analysis was carried out using the Statistical Package for Social Sciences Software (SPSS, vers. 17.0) and with the Statistical Analysis System (SAS, vers. 9.2).

## Results

### Descriptive statistics

Descriptive statistics are summarized in Table 1. Patient survival was comparable for squamous cell carcinoma and adenocarcinoma, with 50% mortality in each group approximately 20 months after diagnosis. Survival of the 12 censored patients was between 54 and 101 months, with a median of 91 months. No gender-specific survival differences were identified. Patients with adenocarcinoma were generally younger and had advanced disease with metastases more often than patients with squamous cell carcinoma. No differences in terms of age, gender, tumour size, nodulus, patient survival or censoring status were noted. The number of patients in the three prognosis groups was determined: those who did not survive 12 months, those with unambiguous prognosis who survived for more than 12 months but less than 60 months, and those who survived 60 months or longer.

**Table 1:** Descriptive statistics for the patients.

	Squamous cell carcinoma	Adenocarcinoma
Gender		
Male	28	28
Female	3	4

Tumour size T		
T1	7	8
T2 T3	13 10	13 8
T4	1	2

Nodal status N		
N0	18	13
N1	7	10
N2	4	7
N3	1	2

Metastasis M*		
M0	31	22
M1	0	10

CD68		
II: 5-30%	2	1
III: 30-80%	29	31

Gas6		
I: < 5%	19	16
II 5-30%	10	14
III 30-80%	2	2

Cox2		
II: 5-30%	3	2
III: 30-80%	28	30

MMP2 *		
II: 5-30%	15	5
III: 30-80%	16	27

Notch3		
II: 5-30%	4	6
III: 30-80%	27	26

Survival status at census		
Dead	23	28
Alive	8	4

	Medians (nonparametric 95% confidence interval)	
	
Age	70 (65-71)	64 (59-69)
Survival time (month)	25 (14-71)	16.5 (11-34)

Demographic data from 63 patients with NSCLC, separated for histology; * marks significant differences in relation to histology.

**Figure 1:**
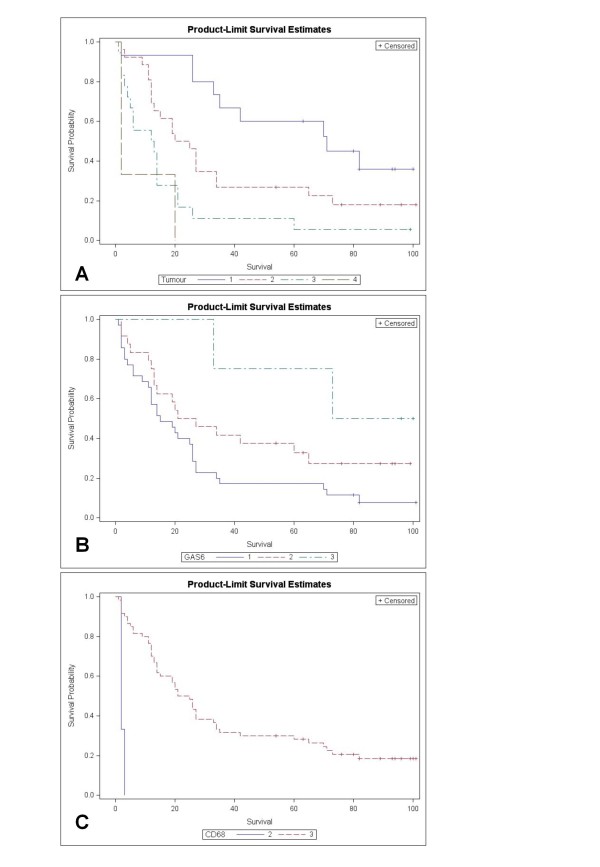
**Product Limit Survival Estimates illustrate the significant impact of T, CD68 and Gas6 (Log rank) on survival of BC**.

Log-rank tests confirmed significant effects on survival with p < 0.001 for T, M, and CD68, p < 0.005 for N, Cox2 and Notch3, and p < 0.05 for Gas6. For the variables T, Gas6 and CD68, Kaplan-Meier curves (Product Limit Survival Estimates) are presented in Figure 1.

Significant (p < 0.05) Spearman correlation coefficients with survival were obtained for T (r_s _= -0.55), N (r_s _= -0.41), M (r_s _= -0.37), and for Gas6 (r_s _= 0.31) and CD68 (r_s _= 0.32), but not for the other proteins or clinical variables (age, gender, histology, MMP2, Cox2, Notch3). Table 2 summarizes the relationship between survival time and TNM status and protein expression, and the AUC to predict a survival of ≤12 and ≥ 60 months for every variable.

**Table 2:** Spearman correlation of survival and AUC for various variables (ability to differentiate between survival of ≤ 12 months and ≥ 60 months).

Variable	*r_S_*	AUC
T	- 0.55	0.82
N	- 0.41	0.80
M	- 0.37	0.64
Gas6	0.31	0.71
CD68	0.32	0.57
Notch3	0.23	0.62
MMP2	0.00	0.50
Cox2	0.25	0.57
ISIR	- 0.63	0.90
Cox	- 0.70	0.94

### Expression patterns of Gas6 and CD68

**Figure 2:**
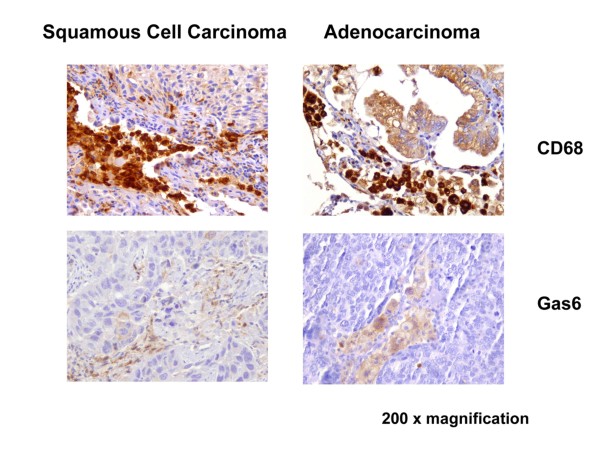
**Immunohistological staining of SCC and AC for Gas 6 and CD68**. Immunohistochemistry for CD68 and Gas6 in representative tumour samples from patients with squamous cell carcinoma (SCC) and adenocarcinoma (AC); 200 × magnification.

Gas6 expression revealed a staining pattern inside the stroma. Positive signals were confined to macrophages, while the tumours themselves were not stained; comparable staining patterns were evident in squamous cell carcinoma and adenocarcinoma (Figure 2). Macrophages expressing CD68 are central to the innate immune response. All tumour samples for squamous cell carcinoma and adenocarcinoma expressed CD68 (alveolar macrophages in the stroma of the tumours, and healthy lung tissue) (Figure 2).

### Integrated Score for an Individual Risk (ISIR)

Assessing risk as a balance of collaborating aggressive and protective variables, the ISIR was calculated as a ratio of weighted sums of significant aggressive (in view of patient survival; from our data T, N, M) and protective (CD68, Gas6) variables. The status of censoring was ignored, but for the present data long survival times were evident for all censored observations. Therefore, the effect of censoring was minimal.

**Figure 3:**
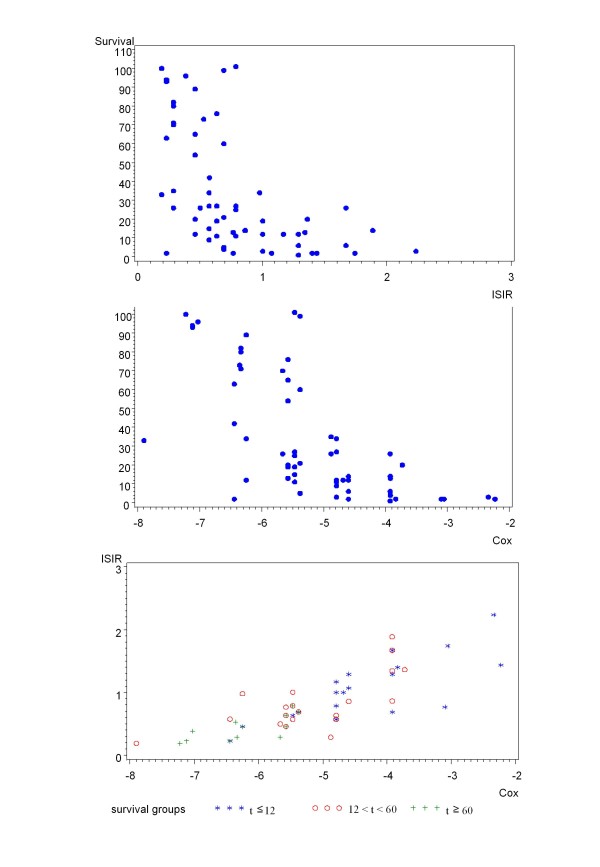
**Relationship between ISIR and Cox**. The respective scatter plots for ISIR (A) and Cox (B), and survival for Cox and ISIR (C), are presented. For the latter, the scatter plot illustrates the monotone dependence between the two classification methods, with those who survive longer in the bottom left and those who survive for a short period in the upper right.

**Figure 4:**
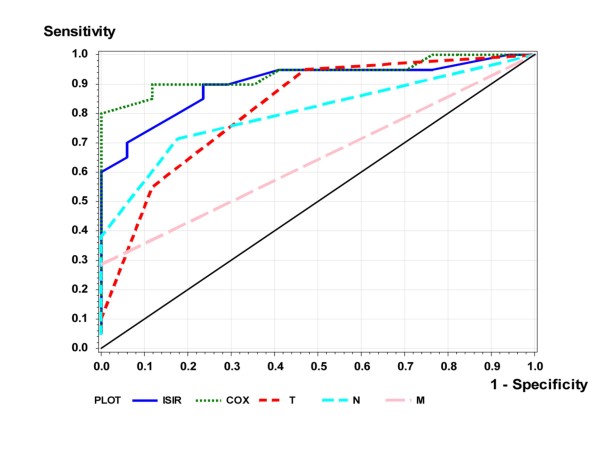
**Cox and ISIR prediction of long-term survival is superior to single markers in patients with NSCLC**. The plot illustrates the ROC with true (sensitivity) and false positive (1-specificity) rates of the introduced formula applied to patients with non-small cell lung carcinoma: theoretical reference line of no discrimination, thin continuous; ROCs using T-, N-, M-status, COX-model and ISIR score (assembling TNM with CD68+Gas6 expression).

The Spearman correlation of ISIR to survival was *r_S_*=-0.63; the absolute value was larger than that for any single variable. Figure 3A demonstrates a scatterplot of ISIR to survival time. In Table 3 the patients are divided into the three groups with clear prognostic assignment according to their individual ISIR-score, i.e. survival ≤ 12, between 12 and 60, and ≥ 60 months. The abilities of ISIR to predict the two survival groups, ≤ 12 and ≥ 60, are presented as ROC curves in Figure 4. The estimated AUC was 0.901. Using the intuitive and handy cut-off value of ISIR = 0.5, the two ISIR classes were defined as "good" if ISIR≤0.5, and as "bad" if ISIR>0.5; 31 of 38 (19 of 21 and 12 of 17) cross validated patients were classified correctly (Table 4).

**Table 3:** Survival of patients assessed with ISIR.

	t ≤ 12	12 > t < 60	t ≥60	
Low risk, ISIR < 0.4	1	3	10	14
0.4 ≤ ISIR ≤ 0.8	7	13	7	27
High risk, ISIR > 0.8	12	8	0	20

	20	24	17	61

Survival time (months) is abbreviated to t. ISIR = (0.55*T/4 + 0.41*N/4 + 0.37*M/2)/3/((0.31*Gas6/4 + 0.32*CD68/4)/2)

**Table 4:** Sensitivities and specificities of the ISIR and Cox methods.

Prognosis not defined 12 >*t *< 60	False positive *t *≥ 60	True Positive *t *≤ 12	True negative *t *≥ 60	False negative *t *≤ 12	Prognosis not defined 12 >*t *< 60
ISIR > 0.5 (n = 42)			ISIR ≤ 0.5 (n = 19)		

19/24	5/17	18/20	12/17	2/20	5/24

Cox > 5.5 (n = 38)			Cox ≤5.5 (n = 24)		

17/25	3/17	18/20	14/17	2/20	8/25

Survival time (months) is abbreviated to *t*.

### Cox regression

The regression parameter *β *= (*β*_1_,.......*β_k_*) in the proportional hazards model (Cox model) was estimated using the method of Maximum Likelihood, with the procedure PHREG from the SAS software. Backward selection was used, and variables remained in the model if the corresponding p-value was less than 0.05. The remaining variables were (together with their estimated regression coefficients): T (0.88), CD68 (-1.60), Gas6 (-0.78), histology (0.68) and Notch3 (-0.80). Perhaps somewhat surprisingly, M and N were not significant in the Cox model. Large values of  indicate short survival.

The term  was replaced by the term Cox we considered to be more instructive. Figure 3B presents a scatter plot of the relationship between Cox and survival time. The Spearman correlation between Cox and survival was -0.70, comparable to that obtained for ISIR. Figure 3C presents a scatter plot of ISIR and Cox. It illustrates the monotone dependence between the two classification methods. Furthermore, as expected, patients with long survival are shown in the bottom left region (indicated with +), and patients with short survival are represented in the upper right region (indicated by *). The ability of Cox to predict the two survival groups, ≤ 12 and ≥ 60 months, was represented as an ROC curve in Figure 4. The estimated AUC was 0.935. Similar to ISIR, Cox was calculated for three risk classes. Here, two observations were classified wrongly (in ISIR it was one, cf. Table 5).

**Table 5:** Patient survival according to Cox classification.

	t ≤ 12	12 < t < 60	t ≥60	
Low risk, Cox < - 6	2	3	11	16
- 6 ≤ Cox ≤ - 4.5	10	17	6	33
High risk, Cox > - 4.5	8	5	0	13

	20	25	17	62

Survival time (months) is abbreviated to t. Cox = 0.88*T + 0.68*Histology - 1.60*CD68 - 0.78*Gas6 - 0.80*Notch3

The cut-off value for Cox was -5.5 (cf. Table 4). Taking this cut-off value, 32 of 38 (14/17 and 18/21) cross-validated patients were represented in the survival classes ≤ 12 and ≥ 60 months, which were classified correctly.

## Discussion

Response to therapy and the corresponding outcome of patients with bronchial carcinoma varies considerably, underlining the requirement for a personalised approach. For the most part, the individual risk profile is estimated from clinical information such as tumour stage. However, rapid advances in biomarker research suggest that tumour aggressiveness and immunological competence of the host must be considered. An increasing number of biomarkers are available for the differentiation of subgroups; the impact of each, whether positive or negative, is predominantly defined by comparisons between patients with a similar TNM status. Considering that several factors influence prognosis and the huge variety of individual constellations, an algorithm to form integrative risk scores is required.

This study confirmed that survival after resection of a non-small cell lung cancer is significantly reduced when the TNM status is improved; in contrast, marked expressions of CD68 and Gas6 as biological markers of the tumour's inflammatory reaction were associated with a favourable outcome. Furthermore, compared with individual markers, integrative models comprising clinical and molecular information provided a higher predictive power to estimate patient prognosis, regardless of whether correlation or regression analysis was used.

In an attempt to characterize the immunological defence of the host, the expression of various proteins involved in numerous physiological pathways related to inflammation and remodelling were analysed. Whether increased expression reflects a favourable outcome is open to debate. For example, expression of Gas6 appears to be beneficial for breast cancer patients but indicates poor prognosis for gastric cancer [8,14,15]. For tumour-associated macrophages (TAM) several functions have been described [16,17]. The observations presented herein are in line with those of Ohri et al. and Kawai et al.; each group observed an improved prognosis related to CD68 expression in NSCLC [18,19]. The expression of Notch was significantly related to longer survival in the Cox model. This agrees with the observation of Dang et al., who described over-expression of Notch in NSCLC [20]. However, it is in contrast to the findings of Konishie et al. They reported that MRK-003 inhibited Notch3 signalling, reduced tumour cell proliferation and induced apoptosis in human lung cancer, indicating that reduced Notch expression may be advantageous to the patient [21]. In summary, indicators of tumour and host biology such as Gas6, CD68 and Notch are helpful for improving the prediction of prognosis after NSCLC, but MMP2 and Cox2 were of no clinical value in the present study. No single factor could provide sufficient predictive power. However, CD68 and GAS6 expression may provide valuable information for an over-all assessment of patient risk.

The increase in information thought to be relevant to a patient's prognosis makes it very difficult to estimate the individual's outcome without condensing all the factors into an integrative risk score. However, research is required into how the best variables for modelling should be selected, and how they should be weighted for optimum prediction of the patient's individual outcome.

Currently, Cox regression is the gold standard for prognostic modelling in cancer [10,22]. However, the selection of potentially influential variables largely depends on the type of optimization and is often unrelated to clinical experience [23]. Cox regression usually results in an abstract algorithm, which is optimised for prediction in a defined collective and can hardly be repeated with distinct cohorts. Whereas the predictive power of any single variable including tumour size was limited, integration of molecular information into a unifying Cox score identified 84% of patients (32 of 38) with a clear prognosis, good or bad. Backward variable selection in a Cox model verified tumour size and histology, and the three molecular markers CD68, Gas6, and Notch3, as relevant factors. TNM had a significant impact on survival using univariate tests, but there was no significant effect of N and M in the Cox model, which is in accordance with the observation of Tsui et al. for renal cell carcinoma. Using a multiple analysis with a Cox proportional hazards model, these authors discovered that tumour stage demonstrated no independent impact on renal cell carcinoma prognosis [24]. In a Cox model to predict survival of patients with gastric cancer, no independently significant relevance of UICC stage was apparent [25].

The ISIR is a simple and easily extendable score. The use of correlation coefficients for selecting and weighting the variables is based on the assumption that any close functional linkage to survival is reflected by significant correlations, negative in the case of shortening survival and positive when indicating longer survival. In fact, a scoring system that uses correlations is able to predict outcome quite as good as a modelling based on Cox regressions. ISIR identified 82% of patients with clearly bad or good prognosis using significant correlations of survival time, with T, N and M being aggressive factors and CD68 and GAS6 being protective factors. By including information relating to molecular markers and clinical stage, the prediction for five year survival was significantly better than that obtained with each single marker, reaching an area under the curve (AUC) of 0.90, which reflects an acceptable predictive power [11,26,27]. Extended gene profiling using Microarrays may not achieve a better outcome prediction; e.g. in breast cancer, microarray performed in a range for AUC of 0.6 - 0.8 [28].

The ISIR score considers the number of variables and the number of possible expression levels. Furthermore, standardisation should help to define general cut-offs that can be transferred to other collectives. However, in the present ISIR, possible close interferences among the variables were not considered. Therefore, the impact of a compound may be overestimated in the case of closely-linked variables with similar functions. It has to be noted that ISIR (and Cox) were evaluated using cross validation. Therefore, the ISIR concerns unbiased estimates of specificity and sensitivity.

The status of genes and proteins must be considered as parts of complex networks rather than of simple linear pathways [29]. Correspondingly, the absolute value of any single marker cannot serve as a reliable estimate of a risk constellation without considering additional interfering and protective influences [26,30]. As a consequence, the expression of biomarkers and clinical information requires integration into comprehensive translational assessments of the patient's risk constellation. The ISIR algorithm and the Cox model use all available information including non-clinical information from genes and proteins, therapeutic interventions and genetic polymorphism or co-morbidities. Therefore, this study presented the ISIR as a novel method for data analysis and applied it to predict disease outcome in a small cohort of patients with bronchial carcinoma. Estimations of the immunological balance of Gas6 and CD68 may supplement other established tumour markers, but their impact on survival will require confirmation in prospective studies.

## Authors' contributions

WK performed statistical analysis of the data and prepared the manuscript. AF basically was involved in the design of the study, evaluated and interpreted the tissue data, together with WK she drafted the manuscript. BK controlled the tissue results, AW provides clinical data, tissue specimen, AT performs immunhistochemistry. UK Conceived and designed the research, worked on the interpretation of data and introduced the conception of ISIR.

All authors read and approved the final manuscript.

## Declaration of competing interests

The authors declare that they have no competing interests.
